# The versatile menu of the nasolabial flap in nasal reconstruction: A defect-based algorithm

**DOI:** 10.1016/j.jpra.2026.07.005

**Published:** 2026-07-10

**Authors:** Tina Deutinger, Evi M. Morandi, Petra Pülzl

**Affiliations:** Department of Plastic, Reconstructive and Aesthetic Surgery, Medical University of Innsbruck, Innsbruck, Austria

**Keywords:** Nasal reconstruction, Local flap, Nasolabial flap, Nasal subunit reconstruction, Facial reconstruction, Defect-based algorithm, Surgical algorithm, Surgical delay, Skin cancer

## Abstract

**Background and objectives:**

The nasolabial flap is a reliable option in nasal subunit reconstruction, but its versatility often remains underappreciated. This study aims to highlight a plethora of different application options of the nasolabial flap for nasal defects, while proposing a reconstructive, defect-based algorithm.

**Patients and methods:**

In a retrospective case series, patients with nasal subunit defects reconstructed using a nasolabial flap between 2014 – 2024 by a single plastic and reconstructive surgeon were included. Data analysis included patient demographics, defect characteristics, nasolabial flap characteristics, perioperative data, and postoperative outcome. Different nasolabial flap applications were categorized into eight variants according to defect analysis by nasal subunit**,** defect depth, and reconstructive complexity. Patient and surgeon satisfaction was rated applying a modified Whitaker scale.

**Results:**

Twenty-three patients (n = 23) were included and classified into the subgroups according to the nasolabial flap applied. In 91.3%, nasal defects were caused by prior skin cancer resection or complications thereof. Defect size ranged from partial (4.3%) and single-subunit defects (78.3%) to multi-subunit defects (17.4%) with varying defect depth between partial- and full-thickness defect. Based on thorough defect analyses, a defect-based treatment algorithm was derived. Variants of the nasolabial flap included transposition and island flap designs, one- vs. two-staged flaps, the nasolabial flap as an enfolding flap or in combination with a turn-over flap with or without concha cartilage grafting. No major complications or flap losses occurred. Postoperative outcome rating showed a high patient satisfaction rate with only one patient demanding a corrective surgery.

**Conclusions:**

The nasolabial flap is versatile and simple, making it highly suitable for facial reconstruction in everyday clinic. Use of the proposed defect-based treatment algorithm enables precise decision making depending on the defect to reconstruct.

## Introduction

The nose plays a pivotal role in facial aesthetics and contributes to essential functions such as breathing and olfaction. It is structured to balance stability and flexibility, with the bony framework providing support and the cartilage and connective tissue allowing movement during facial expressions^1^ The complexity of this structure and the intricate anatomy, comprising various tissues within a small region, complicate surgical reconstruction.^2^ Restoration of nasal symmetry and maintenance of a patent airway are paramount in aesthetic and functional reconstruction of the nose.^3^^,^^4^

Depending on the location, size, and specific tissues involved in a defect, numerous reconstructive options have been described.^5^, ^6^, ^7^, ^8^ Among these techniques, the nasolabial flap (NL flap) stands out as one of the oldest methods used for nasal reconstruction.^9^ Especially for nasal alar reconstruction, the NL flap has become a workhorse flap.^10^ Over recent years though, it has fallen out of favor, largely due to concerns about nasal obstruction and nostril asymmetry.^11^ Despite these drawbacks, the NL flap offers notable advantages for nasal reconstruction, particularly its natural tissue characteristics. The similar texture and color to the adjacent nasal skin enhances aesthetic outcomes, while its donor site is largely inconspicuous, minimizing the visibility of scarring.^5^^,^^12^

Despite its frequent use, the full versatility of the NL flap in nasal reconstruction is usually underappreciated. This study aims to highlight the wide range of applications of the NL flap in reconstructing different nasal defects and subunits. Underscoring its adaptability and effectiveness in addressing nasal defects, we herein present a reconstructive defect-based algorithm further illustrated by case examples. We present the flap's potential in achieving both functional and aesthetic restoration in complex nasal reconstructions.

## Material and methods

### Study design and patients

In this retrospective, single-center study, all patients undergoing nasal reconstruction using a nasolabial flap between 2014 – 2024 performed by a single plastic and reconstructive senior surgeon were included. Inclusion criteria comprised patients presenting with nasal defects ranging from partial subunit defects to complex multi-subunit defects, partial-thickness defects to full-thickness defects, and nasal defects resulting from oncologic skin cancer resection, trauma, or surgical complications such as wound healing disorders. A minimum postoperative follow-up of 3 months was required. Patients undergoing nasal reconstruction without the use of a nasolabial flap or surgery performed by another surgeon were excluded.

Patient records and clinical photographs were reviewed and analyzed for patient demographics, defect characteristics, type of nasolabial flap applied, perioperative data, and postoperative outcome.

All patients signed written informed consent for surgery, data, and image processing. Ethical approval was granted by the ethics committee of the Medical University of Innsbruck (EK number: 1126/2025) and the study was conducted in accordance with the Ethical Principles of the Declaration of Helsinki.

### Defect analysis and classification

For reconstructive planning, all included patients underwent preoperative defect analysis accompanied by standardized photographic documentation and subsequent defect categorization. Defect analysis primarily focused on the involved nasal subunits and defect depth, distinguishing between partial- and full-thickness defects. To additionally evaluate the requirement for structural support by cartilage grafting, the presence or risk of nasal alar collapse was clinically assessed, by direct inspection and during inspiration. Lastly, the need for inner lining reconstruction was addressed depending on the prior analysis of defect size, defect depth, and functional integrity of the nasal framework.

Standardized photographs were routinely obtained for defect analysis, reconstructive planning and documentation. All photographs were taken by a professional medical photographer using a standardized laboratory setup regarding patient positioning and lighting conditions. Standardized photographs included frontal, lateral, oblique (45°), and basal views at rest and during inspiration.

Finally, nasal defects were categorized according to the described defect analysis by nasal subunit, defect depth, and reconstructive complexity and classified into eight distinct groups of nasolabial flap application techniques, as listed in Table 1.

**Table 1:** Variations and technical refinements of the nasolabial flap.

Defect subunit	Defect depth	Defect size (cm)	Defect coverage	Stages of reconstruction	Adjunct/refinement	Complications	Patients (n = 23)	%	Example case illustration
Nasal ala and tip subunit	full-thickness	2.3 × 2.5	Nasolabial flap	2	Turn over flap for inner lining and conchal cartilage, pedicle transected after 3 weeks.	none	2	8.7%	Fig. 2
Total defect ala nasi subunit	Full thickness	2.0 × 1.5	Nasolabial enfolding flap	1	none	none	3	13.0%	Suppl. Fig. 1
Partial defect of ala nasi subunit	Partial thickness	2.5 × 2.5	Nasolabial flap	1	Add conchal cartilage graft if inspirational nostril collapse	none	9	39.1%	Suppl. Fig. 2
Total defect ala nasi subunit	Partial thickness	2.4 × 1.5	Nasolabial flap	2	Add conchal cartilage graft if inspirational nostril collapse. Pedicle transected after 3 weeks.	none	5	21.7%	Suppl. Fig. 3
Inner lining	Partial thickness	4.5 × 3.5	Nasolabial island flap	2	Prepared as island flap for nasal inner lining in nasal reconstruction, combined with forehead flap and conchal cartilage	none	1	4.3%	Suppl. Fig. 4
Partial defect of ala nasi subunit	Full thickness	1.1 × 0.5	Nasolabial flap	1	Turn over flap for inner lining and conchal cartilage	none	1	4.3%	Suppl. Fig. 5
Columella subunit	Full thickness	0.4 × 1.0	Nasolabial flap	2	Conchal cartilage (columella strut graft), pedicle transected after 3 weeks.	none	1	4.3%	Suppl. Fig. 6
Life boat application	Full or partial thickness	n.a.	Nasolabial flap	2	Use as a life boat procedure in total nose reconstruction with forehead flap, pedicle transected after 3 weeks.	Partial tip necrosis, managed conservatively	1	4.3%	Suppl. Fig. 7

### Surgical technique and reconstructive considerations

After thorough defect analysis, the reconstructive approach and flap design was chosen according to the anatomical subunit(s) involved, defect size, and defect depth. Regarding flap width, especially in men hair growth must be assessed thoroughly whereas in women, more tissue can usually be recruited. If necessary, laser hair removal might be a helpful adjunct. Depending on these defect characteristics and the reconstructive reach required, the NL flap was designed as a transposition or island flap with the length-to-width ratio planned individually. Alongside the distinction between partial- and full-thickness defects, the need for structural support by cartilage grafting and for inner lining influences flap configuration. Full-thickness defects requiring inner lining reconstruction were addressed either by enfolding the NL flap alone or by utilizing adjacent local tissue to create a turn-over flap for the inner lining, followed by NL-flap. For cartilage grafting, concha cartilage grafts were routinely harvested and used to restore alar contour and to prevent postoperative nostril collapse if structural instability was present preoperatively. At the same time, full-thickness defects may also be sufficiently reconstructed by an enfolding NL flap without any cartilage grafting if the remaining nasal framework allows for a flap inset under tension avoiding alar collapse.

The decision to perform a one- or two-stage procedure was based on the defect characteristics described above and in consideration of the anticipated aesthetic outcome, and the patient’s preference whenever possible. One-stage procedures were routinely performed in nasal ala subunit reconstruction (Table 1). In these cases, the flap was thinned aggressively especially in the distal two-thirds to minimize bulkiness.^13^ Moreover, the flap pedicle was neatly inset at the alar base to create a natural contour of the alar crease using meticulous suture technique to match the contralateral side.^14^ Our basic two-stage NL flap technique was carried out as previously described in detail elsewhere.^13^ Nasal packing was routinely applied for stabilization of the flap and hematoma prophylaxis in all patients during both the first and second stage surgeries.

### Postoperative outcome assessment

During each patient’s final follow-up visit, professional outcome assessment was routinely performed, and both patient and surgeon satisfaction were documented in the patient’s medical history. For outcome assessment in this study, a modified Whitaker scale^15^ including both patient and surgeon satisfaction ratings was applied.^16^ The modified scale ranging from 1 (defect not corrected, patient not satisfied and desires further correction) to 5 (full correction achieved, patient satisfied) is illustrated in Table 2.

**Table 2:** Rating scale and postoperative outcome for aesthetic evaluation comparing the preoperative finding with the postoperative result modified after Whitaker et al.^1^.

	Surgeons view	N (%)	Patients view documented in medical record	N (%)
**1**	Defect insufficiently corrected	0 (0)	Not satisfied, desires correction	0 (0)
**2**	Only minimal improvement	0 (0)	Not satisfied, sees improvement but desires further correction	0 (0)
**3**	Residual tissue defect	2 (8.7)	Close to satisfied, desires further correction	1 (4.3)
**4**	Small correction warranted	9 (39.1)	Satisfied, no further correction desired	7 (30.5)
**5**	Full correction achieved	12 (52.2)	Very satisfied	15 (65.2)

## Results

### Patients and perioperative data

Twenty-three patients (n = 23) were included for retrospective data analysis. Six patients (6/23, 26%) were male, and 17 patients (17/23, 74%) were female. At the time of reconstructive surgery, mean age was 65.3 (± 14.8, range from 26 to 91) years. Twenty-one patients (21/23, 91.3%) underwent reconstruction of a nasal defect due to previous skin cancer resection or due to complications thereof, while one patient (1/23, 4.3%) was treated following previous trauma affecting the nasal alar subunit and another patient (1/23, 4.3%) was treated due to a columella subunit necrosis after septorhinoplasty. Altogether, defect size ranged from partial subunit defects (1/23, 4.3%), single-subunit defects (18/23, 78.3%), and multiple subunit defects (4/23, 17.4%). While 15 cases showed a partial-thickness defect (15/23, 65,2%), seven cases suffered from a full-thickness defect (7/23, 30.4%). Details on the defect characteristics are listed in Table 1.

Overall, 11 patients underwent reconstruction applying the one-stage NL flap and 12 patients underwent reconstruction using the two-stage NL flap. Mean operation time was 82.4 ± 42.3 min (range from 28 to 211 min) for the first stage surgery and 36.1 ± 26.5 min (range from 10 to 123 min) for the second stage surgery. First stage surgery was performed under local anesthesia in 6 patients (26.1%), under sedation in 7 patients (30.4%) and under general anesthesia in 10 patients (43.5%); second stage surgery was performed under local anesthesia in 12 patients (70.6%), under sedation in 3 patients (17.6%), and under general anesthesia in 2 patients (11.8%). Eleven patients (47.8%) had their first surgery performed in an outpatient clinic setting and 12 patients (52.2%) had an inpatient stay with a mean day of discharge after 1,4 ± 1.9 days (range from 1 to 5 days). No major postoperative complications were reported. We did not experience any flap loss in this series; one patient experienced minor tip necrosis that was managed conservatively (4.3%) . Mean follow-up was 12.5 ± 10.5 months (range from 3 to 30 months).

### Defect-based treatment algorithm and reconstructive patterns

**Fig. 1:**
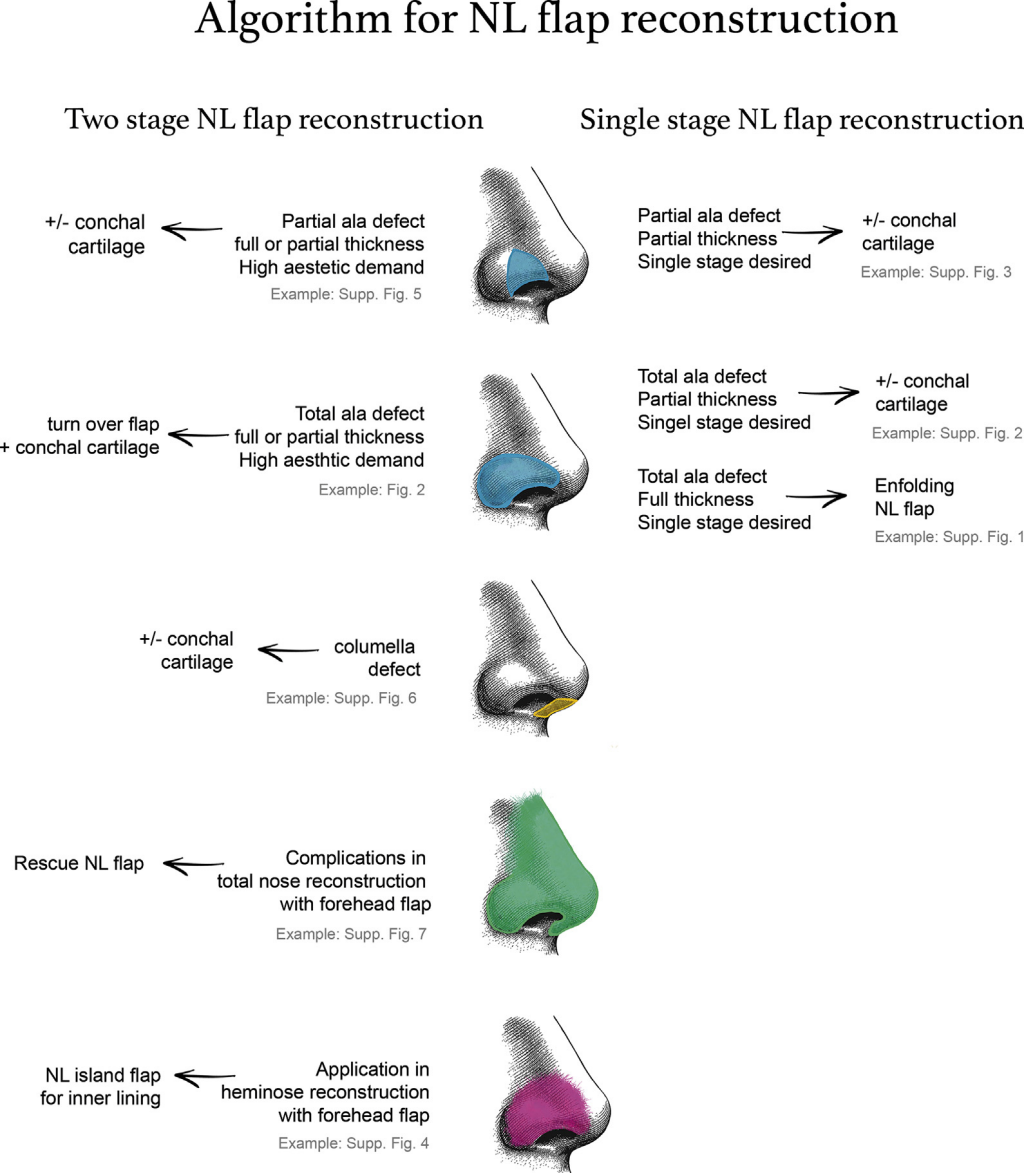
Proposed algorithm for nasal subunit reconstruction using the NL flap. Depending on the defect configuration, one or two stages are employed.

An overview of the different NL flap applications and technical refinements is presented in Table 1. Based on the performed defect analysis, a defect-oriented treatment algorithm was developed to help guide flap design and is presented in Fig. 1.

The algorithm initially builds on defect size by affected nasal subunit(s), followed by differentiation between partial- and full-thickness defect depth. It subsequently incorporates reconstructive requirements including enfolding techniques, turn-over flaps, cartilage grafting, and one- versus two-stage reconstruction. Depending on these defect characteristics, the NL flap is designed as transposition or island flap and combined with adjunctive techniques or flaps.

**Fig. 2:**
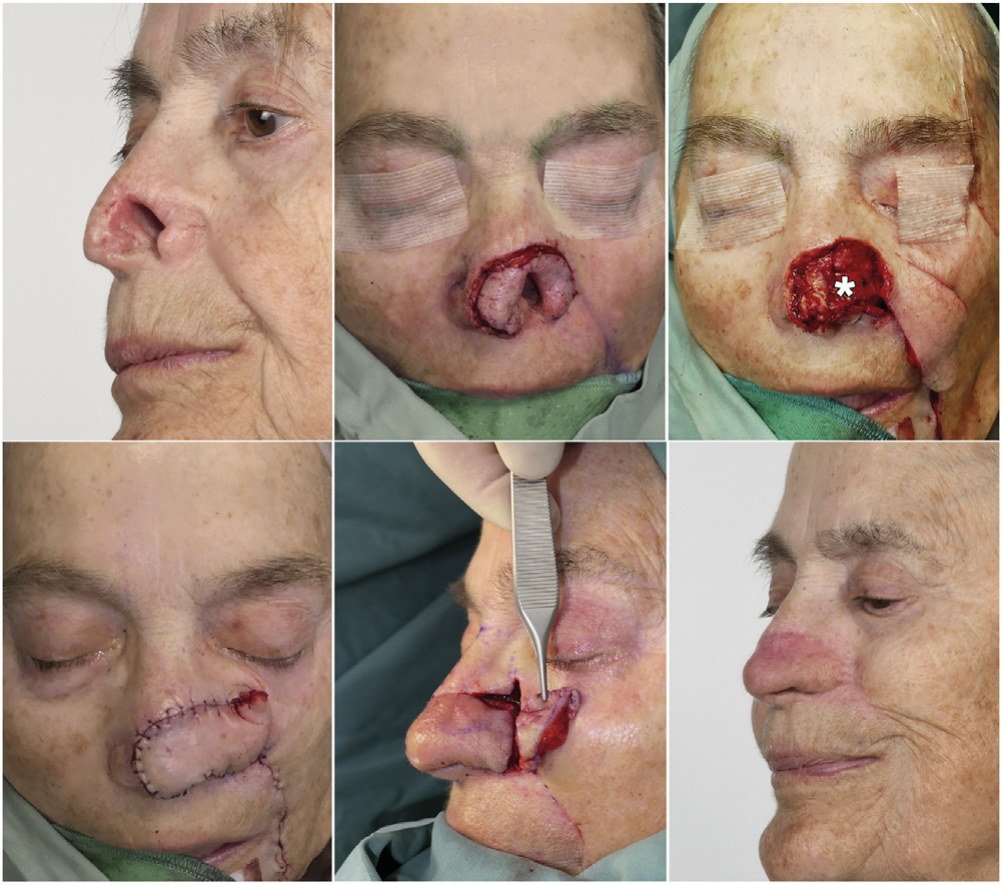
**Two-stage NL flap combined with a turn-over flap and concha cartilage graft for total nasal alar reconstruction of full-thickness defects**. 78-year-old female patient with a full-thickness defect surpassing the nasal ala and thus, involving multiple nasal subunits including the soft triangle after resection of an invasive squamous cell carcinoma (Fig. 2A). An ipsilateral NL flap in combination with a turn-over flap (Fig. 2B, turn-over flap incised, Fig. 4C, turn-over flap lateral and medial pillar united in the middle of the defect -marked by asterisk- and sutured in place) and concha cartilage was performed. The residual skin of the ala and the subunit of the nasal tip was used to create the turn-over flap in her case. A concha cartilage graft was used to stabilize the alar rim, and defect coverage was completed by a transpositional NL flap (Fig. 2D). Pedicle division was performed after 4 weeks (Fig. 2E) along with flap thinning. Postoperative views show an aesthetic and anatomically correct result after 7 months (Fig. 2F). The reconstructed ala appears slightly thicker and might be subjected to a secondary thinning procedure if disturbing the patient.

In this study, full-thickness defects requiring inner lining reconstruction were addressed using NL flaps in combination with turnover flaps and cartilage grafting if structural support was warranted (Fig. 2) or enfolding single stage NL flaps (Supp. Fig. 1). In partial-thickness defects of the nasal ala were primarily reconstructed using one- or two-stage transposition NL flaps (Suppl. Fig. 2,3). In more complex or multi-subunit defects, island pedicled NL flaps with extended arc of rotation were utilized and, if necessary, combined with a forehead flap (Suppl. Fig. 4).

All reconstructive patterns presented within the defect-based algorithm are illustrated by clinical and operative case examples along with operative technique details shortly described in the figure legends (Fig. 2–3, Supplementary Fig. 1–6).

### Postoperative outcome rating

The postoperative outcome was rated by the surgeon and each patient during the final follow-up visit. This was documented in the medical chart. All patients in this series tended to rate their postoperative result better than the surgeon, emphasized by using the higher rating categories only. Of all patients, 95.7% (n = 22) rated their own postoperative result as a 4 or 5, while only one patient complained about a residual contour deformity and desired further correction. This overall postoperative outcome rating result is likely reflecting the patient’s relief from the psychological strain sustained from a centrofacial defect on the nose, especially in cases of two-staged reconstructions. The difficulty of concealing such defects may add to the psychological burden and when choosing a two staged technique, the patient has to deal with a prolonged period of transient deformity due to the flap pedicle.

Regarding the professional judgement of the postoperative outcome, the plastic surgeon was mostly satisfied in 91.3% (n = 21) of cases, while being fully satisfied with the result in 52.2% (n = 12) suggesting a more critical approach to the evaluation of results than the patient group. In nine cases (39.1%), the surgeon offered further minor correction, exhibiting a high demand on optimizing the postoperative aesthetic outcome. An overview of the postoperative outcome rating is shown in Table 2.

## Discussion

The nasolabial (NL) flap, also referred to as melolabial flap, is a historically established flap in facial reconstruction.^9^^,^^17^^,^^18^ Over time, the NL flap has widely become replaced by the forehead flap. While the forehead flap is considered the gold standard for nasal reconstruction, the NL flap has often been limited to be used in partial thickness defects of the lower nose and is most often reserved for elderly patients with significant skin redundancy.^19^ However, this present study demonstrates that this restricted role does not do justice to the versatility of this technique. The NL flap should not only be regarded as an option for small lower nasal defects, but rather as a flexible regional reconstructive system adaptable to various reconstructive requirements.

In general, the NL flap can be designed as advancement, rotation, transposition, or island pedicled flap.^17^ While laterally oriented, partial subunit defects of the nasal sidewall or ala can be reconstructed by advancement and rotation flaps adjacent to the defect, total subunit defects of the nasal ala, columella, or tip require the extended reach of a transposition or island pedicled flap.^17^ As mirrored by our case series, it is a particularly good choice in women that usually have more hairless tissue that can be recruited from the nasolabial fold, but it can easily be used in men as well. If necessary, laser hair removal is a powerful adjunct that can safely provide a permanent removal of hair in the region of interest for various skin flaps.^20^ The flexibility in design for the NL flap further provides surgeons with various strategies to address both partial and full-thickness defects at different subunits of the nose. We herein show two different applications, the one- versus the two-stage NL flap, for the “classical” NL flap indication of partial-thickness defects of the nasal ala (Suppl. Fig. 2 and 3). Depending on local anatomy, defect size, and patient needs, especially the aesthetic aspect of the alar crease may be enhanced by performing a two-stage NL flap (Suppl. Fig. 3).^13^

Full-thickness alar defects may be addressed by creating an enfolding flap for simultaneous inner lining reconstruction. In selected cases, satisfying functional and aesthetic outcomes can be achieved using this simple enfolding flap without cartilage grafting (Supp. Fig. 1). By ensuring adequate tension during inset, the flap stabilizes as the neonasal ala without the risk of nostril collapse. However, for patients who require minimal flap length or the flap length for extensive outer coverage, the NL flap can be combined with cartilage grafts or turn-over flaps from local tissue for a robust inner lining. This combination of techniques makes the flap more adaptable to specific patient needs such as avoiding hair-bearing flap tissue while limiting scar length in young patients (Suppl. Fig. 6) or using additional flap length for defect coverage beyond the ipsilateral nasal ala (Supp. Fig. 7).

The superior vascularity of the NL flap allows for this advanced utility and makes the NL flap suitable for more extensive nasal reconstruction of selected multi-subunit defects or defects requiring extended reach. Perforating branches from the facial artery inferiorly and from the angular artery superiorly anastomose freely and ensure an optimal blood supply allowing flap harvest as either an axial or random pattern flap.^21^, ^22^, ^23^ Even without axial pattern design, the NL flap is described to allow for a length-to-width ratio of up to 6:1.^24^ If the base of the flap is kept at a minimum width of 2 cm, the subdermal plexus and adequate perforators are sufficiently included.^21^ In thin flaps, which may not have captured sufficient vessels, it is worth noting that the perfusion pressure to the distal end of the flap can be compromised.^25^ Especially if the tip needs to be thinned or if the flap exceeds traditional length, delay procedures might be an option.^26^, ^27^, ^28^, ^29^ However, the width of the flap may limit its arc of rotation, which is an important consideration during reconstructive planning. If additional rotation is required, resorting to an island pedicled NL flap allows for a rotation arc well beyond 90°, enabling the flap to cover more distant defects and to create inner linings in extensive nasal reconstruction (Suppl. Fig. 4). In addition, this makes the NL flap also a valuable option for maxillofacial defects, both extra- and intraorally.^30^^,^^31^

Overall, the NL flap offers clear advantages and is not limited to elderly patients with skin redundancy. At all ages, the skin of the nasolabial fold is the most redundant area of the face, which enables efficient wound closure and, moreover, optimal scar camouflage.^17^ In younger patients, the NL flap can serve as an advantageous option due to its ability to use adjacent tissues for natural reconstruction without the need for extensive donor-site scarring. For optimal donor site closure, flap length should always be limited within the boundaries of the nasolabial fold. The surgeon should always be aware not to create ectropium when closing the donor site, preferably shifting the tissue of the cheek in an upward vertical manner.

However, the NL flap has important limitations that need to be acknowledged during defect-based reconstructive planning. In extensive subtotal or total nasal defects, the NL flap alone may not provide sufficient tissue for adequate coverage, and the forehead flap remains the gold standard. The forehead flap as well as free tissue transfer offer superior tissue availability, and the ability to restore larger aesthetic subunits at once. Similarly, in patients with compromised regional tissue or vascularity, local reconstructive options can become limited. Nevertheless, the NL flap may serve as a valuable adjunct to regional or free flap techniques, particularly for refinements of the alar region, inner lining reconstruction, correction of residual defects, or as a salvage option as illustrated in Suppl. Fig. 7. The NL flap should not be regarded as a replacement, but rather as part of a reconstructive spectrum selected according to defect characteristics and regional tissue availability.

In conclusion, this work reinforces the notion that the NL flap remains a valuable technique in nasal defect reconstruction applicable in everyday practice. In addition, the describe a defect-based treatment algorithm that offers a systematic framework of NL flap variations according to reconstructive demands. Limitations of the study include the sample size and the retrospective character of the work.

## Data availability statement

The data supporting this study are available from the corresponding author upon reasonable request.

## Funding statement

This study did not receive any financial funding.

## Ethics approval statement

Ethical approval was granted by the ethics committee of the Medical University of Innsbruck (EK number: 1126/2025).

## Patient consent statement

Written informed consent was obtained from all patients for surgery, data, and image processing.

## Declaration of competing interest

The authors declare that there is no conflict of interest that could be perceived as prejudicing the impartiality of the research reported.

## Appendix Supplementary materials

Image, application 1
