# Supplementary material for: The versatile menu of the nasolabial flap in nasal reconstruction: A defect-based algorithm
Source: JPRAS Open. 2026 Jul 10;51:494–503. doi: 10.1016/j.jpra.2026.07.005 (PMC13453591; doi:10.1016/j.jpra.2026.07.005)
Supplement: Supplementary file 1 [file mmc1.docx]

**Supp. Fig. 1: One-stage NL flap for nasal alar reconstruction of full-thickness defects.** 57-year-old female patient with an extended full-thickness lateral alar defect including multiple facial subunits caused by basal cell carcinoma resection (Supp. Fig.1A). A combination of reconstructive methods with a bilateral bullhorn lip lift for upper lip reconstruction and alignment along with a one-stage ipsilateral NL flap was chosen. The NL flap was performed with a length-to-width-ratio sufficient to be folded over (Supp. Fig. 1C) after primary aggressive flap thinning for reconstruction of the alar rim (Supp. Fig. 1D). Well balanced tension during flap inset and a postoperative nasal tamponade for 2 weeks ensured nostril patency and stability without the use of a cartilage graft. The flap decreased in bulkiness after 3 months of conservative measures such as scar massage and lymphatic drainage and the patient did not seek further surgical debulking. Postoperative pictures after 12 months are shown in Supp. Fig. 1D-F and illustrate very well, that the endonasal part of such flaps should be subjected to routine dermatologic follow ups especially in skin cancer patients as the consist of facial skin prone to the development of secondary lesions.

**Supp. Fig. 2:** 73-year-old female patient left with a partial thickness defect of the left nasal ala after basal cell carcinoma resection. The inner lining and most of the alar cartilage are preserved. The reconstruction used concha cartilage graft from the ipsilateral side (Supp. Fig.2A, asterisk), and a classical ipsilateral one-stage NL flap with a 3:1 length-to-width-ratio to prevent nostril collapse. Intraoperative views from lateral (Supp. Fig. 2B) and inferior (Supp. Fig. 2C) show the immediate result. Postoperatively, symmetric nasal ala is evident in frontal view (Supp. Fig. 2D). A slight contour elevation as evident in the oblique and inferior views (Supp. Fig. 2E, F) was not disturbing the patient but could otherwise be subjected to a secondary thinning of the flap.

**Supp. Fig. 3: Two-stage NL flap for nasal alar reconstruction of partial thickness defects with concha cartilage graft.** 59-year-old female patient with a partial thickness defect of the left nasal ala after resection of a basal cell carcinoma. Inner lining was preserved. The defect was not confined to the alar subunit but involving a small part of the adjacent cheek (Supp. Fig. 3A). The patient reported difficulties breathing through the left nostril due to contracted and collapsing ala. A two-stage approach with a concha cartilage for alar rim support (Supp. Fig. 3B, asterisk marks cartilage graft) and an ipsilateral NL flap with a 5:1 length-to-width-ratio (Supp. Fig. 3C) was conducted. Due to the configuration of the defect and the high pivot point, flap inset determined use (Supp. Fig. 3D) and later division (Supp. Fig. 3E, F) of a flap pedicle, carried out after 3 weeks. Supp. Fig. 3G-I show the postoperative result 3 months after pedicle division.

**Supp. Fig. 4: Nasolabial island flap for nasal inner lining reconstruction.** 81-year-old male patient with an extensive defect of the entire nasal bridge, nasal tip and left ala, part of the right ala, the right cheek, and the cranial part of the columella (Supp. Fig. 4A), following wide resection of a recurrent basal cell carcinoma. The inner lining was constructed with a NL flap with a ratio 5:1 length-to-width-ratio (Supp. Fig. 4B) turned inside out (Supp. Fig. 4C, arrow). Forehead flap reconstruction was carried out (Supp. Fig. 4D, E). According to Menick’s technique, aggressive flap thinning of the forehead flap and, in this case, also the NL flap was carried out after 6 weeks along with introduction of concha cartilage to add stability (Supp. Fig. 4F). After 5 weeks, the pedicle was divided. Supp. Fig. 4G-I show the postoperative result after 3 months.

**Supp. Fig. 5: Two-stage NL flap combined with a turn-over flap and concha cartilage graft for partial alar reconstruction of full-thickness defects**. 65-year-old female patient with a full-thickness defect of the caudal left ala resulting in a high vertical notch and collapsing residual alar tissue after basal cell carcinoma resection and application of full thickness skin graft (Supp. Fig. 5A-C). The turn-over flap for reconstruction of the inner lining was created by incising the border of the well healed skin graft (Supp. Fig. 5D, asterisk), creating the new inner lining as a turnover flap. A pedicled (Supp. Fig. 5E, arrow) NL flap was applied to create the outer lining and cover the concha cartilage graft. During pedicle transection, flap debulking was conducted as well (Supp. Fig. 5F). Supp. Fig. 5G-H show the postoperative result after 9 months.

**Supp. Fig. 6: Two-stage NL flap for columellar reconstruction.** 26-year-old male patient with a history of a Serratia infection after rhinoplasty, leading to scarring and resulting in massive shortening of the columella, thus compromising the nasal tip and distorting the right ala (Supp. Fig. 6A-C). To cover the defect resulting from scar incision (Supp. Fig. 6D, arrow), a two-stage pedicled nasolabial was performed to reconstruct the columella (Supp. Fig. 6E, asterisk). After four weeks, pedicle transection was carried out and excess tissue was removed (Supp. Fig. 6F). A minor skin retraction was secondarily corrected with additional concha cartilage used as a small columella on lay graft. Postoperative pictures show the result after 6 months (Supp. Fig. 6G-I)

**Supp. Fig. 7: Two-stage NL flap as a complication management lifeboat in nasal reconstruction.** 59-year-old, heavily smoking female patient with a near total nasal defect after massive basal cell carcinoma (Supp. Fig. 7A, B) undergoing nasal reconstruction with a forehead flap, pivot-flap and rib cartilage scaffold. She had partial flap necrosis after thinning the forehead flap ins second stage (Supp. Fig. 7C, D). Correction with a two-stage NL flap was conducted (Supp. Fig. 7E). Again, flap perfusion was compromised (Supp. Fig. 7F, asterisk) Local infection caused partial resorption of the rib cartilage graft, reducing tip projection. Salvage revision surgery was carried out with debridement, concha cartilage on lay graft and NL flap from the contralateral side (Supp. Fig. 7G). The postoperative result is shown in Supp. Fig. 7H, I, emphasizing the projection of the reconstructed nose despite all complications and the power of the NL flap as a salvage procedure.
